# Superior colliculus modulates the compulsion in a model of addiction with foot-shock

**DOI:** 10.1038/s41398-026-04103-5

**Published:** 2026-05-21

**Authors:** Meng-Die Yang, Jian-Guo Chen, Fang Wang, Ning Wu, Jin Li, Li-Hong Long, Rui Song

**Affiliations:** 1 State Key Laboratory for Diagnosis and Treatment of Severe Zoonotic Infectious Diseases, Department of Pharmacology, School of Basic Medicine, Tongji Medical College, Huazhong University of Science and Technology, Wuhan, 430030 China; 2 Academy Military Medical Sciences, Beijing, 100850 China; 3 State Key Laboratory of National Security Specially Needed Medicines, Beijing, 100039 China

**Keywords:** Epigenetics and behaviour, Addiction

## Abstract

Compulsive drug use despite negative consequences is a core addiction feature and key therapeutic target. Animal models utilize footshock to screen for mice exhibiting compulsive-like addiction traits. Following the administration of aversive stimuli, compulsive animals persist in drug-seeking, suggesting that addicted individuals may have impaired innate defensive responses, thereby exacerbating addictive behaviors. However, little is known about the neural mechanisms behind this behavior. The superior colliculus (SC), a multisensory integration hub, plays a crucial regulatory role in innate fear and defense. This study employed an optical intracranial self-stimulation (oICSS) addiction-like model. Using footshock to screen for mice with compulsive-like behavior, fiber photometry recordings revealed significant differences in neuronal activity within the SC. Specifically, SC neurons in compulsive-like mice showed significantly lower responses to footshock stimuli compared to non-compulsive mice. Subsequently, chemogenetic inhibition of SC neuronal activity in non-compulsive mice significantly reduced their resistance to footshock, inducing a compulsive-like state. Conversely, chemogenetic activation of SC neurons in compulsive-like mice significantly decreased their oICSS behavior. These findings indicate that mice exhibiting compulsive-like addiction behavior, identified through footshock, exhibit significant functional abnormalities in SC neurons. The SC is implicated in regulating compulsive addictive behaviors, providing novel insights into the mechanisms of compulsivity and identifying a promising new target for addiction intervention.

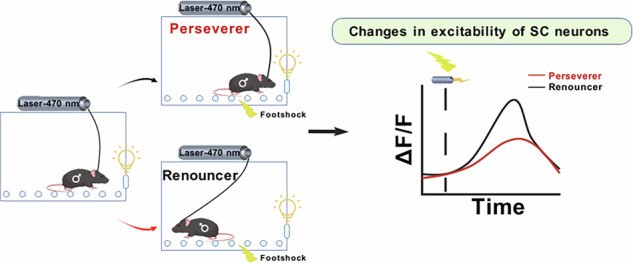

## Introduction

Reckless compulsive drug use behavior is a core feature of addiction, one of the intractable characteristics of addiction, and a key issue in addressing addiction treatment [1, 2]. Current research focuses on screening for addicted animals with compulsive behavioral traits by employing footshock in animal models [3–6]. The fact that compulsive animals did not choose to give up their drug use behavior when given a negative stimulus suggests that the instinctive defenses of addicted individuals may be impaired, which in turn promotes addictive behavior. However, the neural mechanisms behind their behavior are unclear.

The superior colliculus (SC) is a key layered structure in the midbrain, that plays a central pivotal role in survival-related instinctive defense behaviors and multisensory integration for environmental perception [7–9]. Based on neuronal distribution patterns, connectivity, and functional properties, the alternating layers of neurons and fibers in the SC of all vertebrates can be roughly subdivided into either the superficial, intermediate, and deep layers in mammals, or in the optic tectum (OT) of non-mammals, into the superficial, central, and periventricular layers [10–12]. It was found that SC superficial (especially optic layer) neurons are susceptible to potential visual threats such as rapidly approaching objects [13, 14]. The detected threat signals are rapidly transmitted to the deeper layers of the SC, activating specific groups of “defense neurons” [15]. These neurons trigger rapid, automated defense responses, including freezing immobility, flight, or evasive maneuvers [16, 17], by projecting strongly to specific subregions of the periaqueductal gray matter (PAG) (e.g., dorsolateral PAG) [18, 19]. The deeper layers of the SC (especially the middle gray matter layer) are rich in multisensory neurons that respond simultaneously to visual, auditory, and somatosensory stimuli [20–25]. The integration follows the core principles of 1) spatial coherence (stimuli need to come from the same location); 2) temporal coherence (stimuli need to be nearly synchronized); and 3) superadditivity effect (multisensory combinations of responses are much more than the sum of the single senses), which significantly enhances the probability of detection of an event, the accuracy of localization, and the speed of response [15, 20]. This efficient integration is critical for quickly orienting attention (e.g., turning to sudden sounds or flashes of light) and for providing more reliable environmental information for defense decisions [10, 23, 26, 27].

The SC has traditionally been viewed as a key midbrain structure for processing visual orienting and defense. However, studies have found that it is also a central hub for associating environmental visual cues with drug reward [28–32]. The SC receives and efficiently processes visual information with motivational salience (e.g., drug-related scenes or objects) [33, 34]. These cues, which are repeatedly paired with drugs, are detected by the SC and specifically activate dopamine neurons in the ventral tegmental area (VTA) through direct glutamatergic projections from its deep neurons [35]. This activation drives dopamine release in target regions such as the nucleus accumbens [35, 36], mimicking the reward effect of the drug itself or generating anticipatory reward signals that trigger strong feelings of craving and drug-seeking behavior [37]. Chronic drug exposure may lead to enhanced SC responsiveness to drug-related cues and plastic changes in SC-VTA pathway function (e.g., sensitization), causing these cues to acquire abnormal salience, persistently “hijacking” the reward system and significantly increasing the risk of relapse [38–40]. Thus, the current study suggests that the SC is a key node for visual information transmission, which is specifically involved in the regulation of reward behavior through neural connections with the VTA [30]. Based on the above information, we hypothesize that SC may be involved in regulating the formation and maintenance of addiction-related compulsive behaviors. However, there is still a lack of systematic research evidence on whether SC is involved in addiction-related compulsive behaviors by mediating negative reinforcing stimuli.

Optogenetics is widely regarded as the most transformative breakthrough in 21st-century neuroscience [41]. This technique enables real-time manipulation of neuronal activity by expressing light-sensitive proteins in targeted cells, which respond to specific wavelengths of light [42]. Studies suggest that the dopaminergic reward center within the mesolimbic pathway serves as the primary driver of addiction, with dopamine (DA) neurons constituting the most critical neural substrate mediating its functional effects [43–45]. Therefore, utilizing optogenetics enables the precise, real-time manipulation of DA neurons, combined with self-administration paradigms. This integrated approach provides an ideal animal model—optical intracranial self-stimulation(oICSS)—for elucidating the pathogenic mechanisms underlying addiction [46]. This model enables experimental animals to trigger the optogenetic activation of specific neural circuits through their voluntary behaviors. The self-administration of light stimulation targeting VTA DA neurons recapitulates the core features of addiction, faithfully modeling its cardinal behavioral manifestations: compulsive seeking [3, 5] (persistent behavior despite adverse consequences), enhanced motivation [47] (excessive effort expenditure to obtain stimulation), and post-abstinence reinstatement [48, 49]. Therefore, the oICSS model serves as a behavioral paradigm for addiction research, facilitating the understanding and elucidation of the neural mechanisms underlying the core features of substance addiction.

In this study, an addiction-like model of oICSS was used to screen mice with compulsive behaviors by footshock. Significant differences in neuronal responsiveness of SC’s were found by the technique of fiber-optic recording, with SC neurons in compulsive mice being significantly lower than those in noncompulsive mice when given foot-shock stimulation. The activity of neurons in the SC of specifically noncompulsive mice was converted to a compulsive-like state by chemogenetic methods that significantly enhanced resistance to footshock; in contrast, the neuronal activity of neurons in the SC of specifically activated compulsive mice significantly reduced intracranial self-administration of light behaviors in the mice. The above results suggest that the SC neurons in the brain, which were screened into compulsive addicted mice by footshock, have significantly abnormal functions and are involved in the regulation of addictive compulsive behaviors, which provide new ideas for the revelation of the mechanism of compulsive behaviors, and new targets for the intervention and treatment of addictions.

## Materials and methods

### Animals

A total of 105 male C57BL/6 J mice were used in this experiment. All animal procedures were approved by the Institutional Animal Care and Use Committee under permission No. IACUC-DWZX-2023-669 was performed strictly per the Guidelines for the Care and Use of Laboratory Animals. C57BL/6 J mice were procured from SPF (Beijing) Biotechnology Co., Ltd. Male adult mice (8 - 10 weeks old) were housed in groups of 5 per cage and maintained on a 12 h light/dark cycle (with lights on from 7:00 AM–7:00 PM) with ad libitum access to food and water.

### Viruses

#### For optical intracranial self-stimulation(oICSS) experiments

rAAV-hEF1α-DIO-hChR2(H134R)-mCherry-WPRE-PA (serotypes: AAV2/9, titers: ≥1 × 10^13 ^μg/mL, Taitool Bioscience Co., Ltd, Shanghai, China); rAAV-mTH-NLS-CRE-WPREs (serotypes: AAV2/9, titers: 5.27 × 10^12 ^μg/mL, BrainVTA Co., Ltd., Wuhan, China). They were mixed in a 1:1 ratio, and 200 nl was injected bilaterally.

#### For Fibre photometry recordings experiments

rAAV-syn-JRGECO1α (serotypes: AAV2/9, titers: 3.11 × 10^12 ^μg/mL, OBiO Co., Ltd, Wuhan, China, volume: 200 nL /unilaterally).

#### For specific regulation SC experiments

rAAV-hSyn-hM4D(Gi)-EGFP (serotypes: AAV2/9, titers: 5.42 × 10^12 ^μg/mL, Brain Case Co., Ltd, Wuhan, China, volume: 150 nl /side); rAAV-hSyn-hM3D(Gq)-EGFP (serotypes: AAV2/9, titers: 5.42 × 10^12 ^μg/mL, Brain Case Co., Ltd, Wuhan, China, volume: 200 nl /side);

### Drugs

Xylazine was purchased from Shanghai Macklin Biochemical Co., Ltd, and CNO was purchased from America APExBIO Co., Ltd (Cat. No A3317).

Ketamine was received from the Ministry of Public Security.

### Locomotion test

Mice were habituated to the locomotor detection chamber for 30 min for 2 days. Subsequently, based on the average moving distance of the mice within 30 min over a period of 2 days, they were randomly and evenly divided into three groups to ensure that there was no statistically significant difference in activity levels among the groups. On test day, the mice were placed in the locomotor detection chamber, and locomotor activity was recorded for 60 mins. Data acquisition was conducted using Anilab version 8 for Locomotor software and analyzed using the Anilab data analysis system (Anilab Instruments, China).

### Hot plate test

Preheat the heating plate to 55 °C ( ± 0.5 °C). Place the mice in the experimental cage 30 min in advance, to allow them to adapt to the environment. During the actual experiment, place the mice on the metal surface of the heating plate, start timing, and carefully observe the mice’s behavior until they show a painful response (jumping or licking their hind paws). Then stop timing and record this time. This period is the baseline thermal pain threshold of the mice [50]. The cut-off time for the test was 30 s to avoid tissue damage.

### Virus injection and implantation

The surgery was performed under anesthesia using 10 ml/kg of a solution containing xylazine (12.5 mg/kg; i.p.) and ketamine (100 mg/ kg; i.p.). The degree of anesthesia was confirmed by pinching the toe or tail, once in a state of anesthesia (lack of response to toe pinch and absence of eyelid reflex), we performed the microinjection surgery. During and after the surgery, mice were placed on a heated pad to prevent post-operative hypothermia. To maintain eye lubrication, the ophthalmic ointment was applied to reduce post-operative discomfort caused by eye dryness. Keep the skull horizontal between bregma and lambda.

#### For optical intracranial self-stimulation(oICSS) experiments

The mixed rAAV constructs were injected unilaterally into the VTA at the following coordinates relative to the posterior fontaner: AP − 3.2 mm, ML ± 0.5 mm, and DV − 4.2 mm (DV measured from the dural surface). Injections (rAAV-hEF1α-DIO-hChR2(H134R)-mCherry-WPRE-PA, rAAV-mTH-NLS-CRE-WPREs) were performed at 30 nL/ min using a WPI Nanoliter 2000 syringe (WPI, Florida, USA). The injection volume was 200 nl for each side. The injector remained in place for an additional 10 mins. Subsequently, a single optic fiber cannula (OD = 200 μm, 0.37 NA; ThinkerTech Nanjing Biotech, Nanjing, China) was implanted in the right VTA using the same coordination as the mixed rAAV injection described above.

#### For Fibre photometry recordings experiments

The rAAV-syn-JRGECO1α were injected unilaterally into the SC at the following coordinates relative to the posterior fontaner: AP − 3.7 mm, ML − 0.8 mm, and DV − 1.8 mm (DV measured from the dural surface). Injections were performed at 30 nL/ min using a WPI Nanoliter 2000 syringe (WPI, Florida, USA). The injection volume was 200 nl unilaterally. The injector remained in place for an additional 10 mins. Subsequently, a single optic fiber cannula (Cat. No. 62003, OD = 200 μm, 0.37 NA; RWD Life Science, Shenzhen, China) was implanted in the right SC using the same coordination as the rAAV injection described above.

#### For specific regulation SC experiments

rAAV-hSyn-hM4D(Gi)-EGFP, rAAV-hSyn-hM3D(Gq)-EGFP was bilaterally infused into the SC: AP − 3.7 mm, ML − 0.8 mm, and DV − 1.8 mm (DV measured from the dural surface). Injections were performed at 30 nL/ min using a WPI Nanoliter 2000 syringe (WPI, Florida, USA). The injection volume was 200 nl for each side. The injector remained in place for an additional 10 mins.

Buprenorphine (0.1 mg/kg, s.c.) was administered after surgery to relieve pain; mice were allowed 2-weeks recovery before training.

### Optical intracranial self-stimulation (ICSS) experiment training

#### Acquisition

The chambers (AniLab Software and Instruments) were equipped with two nosepoke holes: one was active nosepoke, and the other was inactive nosepoke. The mice learned to self-stimulate dopaminergic neurons in the VTA infected with the mixed rAAV (ICSS) for 11 consecutive days. Each of the 11 acquisition sessions lasted 60 mins. In each oICSS session, mice could respond to the active poke, resulting in VTA stimulation. Responding to the inactive poke had no consequences. During the first 5 sessions, a single response in the active nosepoke (termed fixed-ratio one or FR1) resulted in a 5 s illumination of a cue light. Another 3 s (473 nm) laser stimulus (15-ms pulse width at 20 Hz) was applied simultaneously. A 10 s time-out followed the rewarded poke, during which pokes had no consequences but were still recorded. Next, a FR2 (sessions 6–8) and a FR3 (sessions 9–11) were introduced.

#### Progressive ratio test

After the acquisition sessions, the mice underwent a progressive ratio test to measure their motivation for the laser stimulus, and the test session lasted for 6 h. The breakpoint was the cumulative number of nose pokes that the mice performed before it ceased poking after 60 min without receiving laser stimulus. The reinforced schedules were according to the following progression: 1, 2, 4, 6, 9, 12, 15, 20, 25, 32, 40, 50, 62, 77, 95, 118, 145, 178, 219, 268, 328, 402, 492, and 603.

#### Punishment test

After 15 days of oICSS training, the mice received 1 h punishment for 3 consecutive days. The FR3 training in the 3 days before the punishment was the baseline behavior. The punishment session was conducted under the same conditions as the baseline session, using FR3 training. A footshock (500 ms, 0.2 mA) was delivered every three laser stimulation events (the shock was delivered simultaneously with the laser stimulation).

### Fiber photometry

By leveraging the strict correlation between changes in calcium ion (Ca2 + ) concentration and neuronal activity, using special fluorescent dyes or protein fluorescence probes, the concentration of calcium ions within neurons can be expressed as fluorescence intensity and captured by the optical fiber recording system, thereby achieving the goal of detecting neuronal activity. The sampling rate was set to 100 Hz. We used single-channel fiber photometry system provided by Thinker Tech (Nanjing, China). The 488-nm excitation light from a semiconductor laser (Coherent, OBIS 488 LS, tunable power up to 50 mW) was reflected by a dichroic mirror with a 452- to 490-nm reflection band and a 505- to 800-nm transmission band (Thorlabs, MD498) and then coupled to a fiber by an objective. The emission fluorescence was detected by a photomultiplier tube (Hamamatsu, H10720-210) after filtering by a red fluorescent protein (JRGECO1α) bandpass emission filter (Thorlabs, MF525-39). The analog fluorescence data were streamed into a DAQ card (National Instruments, USB-6001), and the signal was filtered by a low-pass filter (cutoff frequency, 30 Hz) and saved on the computer. The intensity of the excitation light at the distal end of the optical fiber was adjusted to 40–60 μW.

During oICSS, calcium changes in mouse neurons were recorded by three color optical fiber photometry systems. During oICSS experiments, calcium signal acquisition and the AniLab Software, an operant behavior program, were initiated simultaneously. The laser stimulus time stamps during the formation phase and the footshock recorded by AniLab Software were used to synchronize events in the photometry signal. JRGECO1α was excited with 580 nm light, and signals at 405 nm were also recorded.

### Clustering analysis

Clustering analysis was performed with Origin. Before clustering, the eight variables of each mouse were normalized. The ratio of the footshock test values over three days to the average of the values in the three days prior to the shock, multiplied by 100%. The six variables included active nosepokes, futile nosepokes, and laser-stimulated obtained from the last two punishment sessions (P2 and P3). Futile nosepokes were defined as active nosepokes during the 10 s timeout, reflecting the level of mice impulsion. After standardizing the 6 indicators, hierarchical clustering analysis was conducted using the average linkage method based on Manhattan distance. The clustering results showed that the animals were divided into two distinct clusters: Cluster I (shock-resistant) and Cluster II (shock-sensitive). The results were visualized through a heatmap with column dendrogram. t-SNE is a dimensionality reduction technique used to represent high-dimensional data sets in a two-dimensional or three-dimensional low-dimensional space, thereby enabling their visualization. The nonlinear dimensionality reduction algorithm used in t-SNE experiments is highly suitable for reducing high-dimensional data to two or three dimensions and visualizing the data.

### Data analysis and statistics

For fiber optic recording data analysis, MATLAB 2017 was used, and the baseline correction strategy was adopted. Polynomial Fitted correction was applied to mitigate photobleaching effects, and data were segmented based on behavioral events within individual trials. For population analysis, each mouse served as a data point. After subtracting noise signals, data from the neuronal excitability change experiment during self-administration were smoothed with a moving average filter. ΔF/F traces were obtained using a script provided by Thinker Tech, calculated as (F-F_0_)/F_0_, where F_0_ is the baseline fluorescence signal averaged over a control time window. ΔF/F values were presented with heatmaps and average plots, with shaded areas indicating SEMs. Raw signals were adjusted with a flat baseline and then converted to ∆F/F by dividing by the mean of the original signal. The fluorescence data were collected from perseverer and renouncer mice in each test for 1 h. Area under the curve (AUC) was calculated as the integral between 0 and 3 s.

Statistical analyses were performed with SigmaStat 3.5. One-way analysis of variance (ANOVA), two-way ANOVA, or Student’s t test was used to analyze the data. A nonparametric test was used if the data did not meet a Gaussian distribution. The Bonferroni test was used for post hoc analysis after ANOVA. Data were presented as mean ± SEM or mean ± interquartile range. Statistical details were presented in the figure legends. Significance was defined as *P < 0.05, **P < 0.01, and ***P < 0.001.

Animal group sizes were chosen based on extensive previous experience with the animal models used. The group size is the number of independent values (individual animals), and statistical analysis was done using these independent values. No data points were excluded from the analysis in any experiment. The sample size was sufficient to support all planned analyses. All investigators involved in data measurements and analysis were blinded to group assignments until all measurements were completed and the dataset was locked for analysis. To validate the use of parametric statistics, we performed a Shapiro Wilk Test for data normality evaluation and Levene’s test for homogeneity for between-subject ANOVA.

## Result

### Compulsive self-stimulation of dopamine neurons

**Fig. 1:**
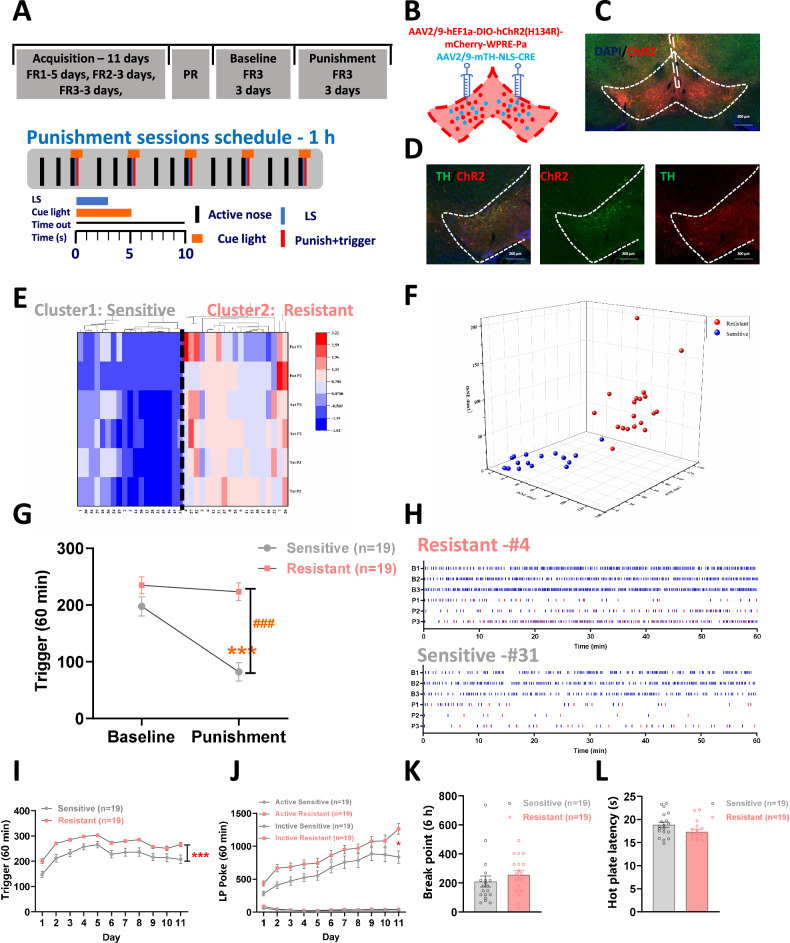
A subpopulation of mice displayed addictive compulsive behavior. **A** Experimental timeline for identifying mice with oICSS compulsive behavior. **B**, **C** Schematic of virus injection sites and optic fiber implantation sites. Scale bars, 200 μm. **D** ChR2-mCherry and a coronal slice colabeled with tyrosine hydroxylase (TH). Scale bars, 200 μm. **E** Hierarchical clustering was performed based on the homogenization of different parameters of penalty sessions 2 and 3 (P2 and P3) of oDASS. Red and green arrowheads indicate examples presented in (H). **F** t-SNE three-dimensional representation of clusters of resistant (cluster 1) and sensitive (cluster 2) in oICSS. **G** Resistant and sensitive laser stimulation obtained from the baseline and the punishment session. Resistant had more laser stimulation compared with sensitive. Two-way analysis of variance (ANOVA) revealed a statistically significant interaction effect, F_1,75_ = 10.635, **P = 0.002, Holm-Sidak post hoc analysis, ###P < 0.001 for sensitive versus resistant in punishment, ***P < 0.001 for punishment versus baseline in sensitive; n = 19, respectively. **H** Raster plots for laser stimulation (blue lines) and punishments (red lines) in baseline and punishment sessions of a Resistant. **I**, **J** Number of active nosepokes, inactive nosepokes, and laser stimulation from acquisition sessions. Two-way ANOVA showed significant main effects on time(F_10,180_ = 52.59, ***P < 0.001) and group (F_1,18_ = 8.649, **P = 0.0087)for Trigger. Two-way ANOVA showed significant main effects on time (F_10,396_ = 16.73, ***P < 0.001) and group(F_1396_ = 46.62, ***P < 0.001) for active nosepokes. Two-way ANOVA showed significant main effects on time(F_10,396_ = 3.394, ***P < 0.001) for inactive nosepokes, and showed no significant effects on groups(F_1,18_ = 0.6956, P = 0.4152). n = 19, respectively. Data are presented as mean values ± SEMs. **K** Breakpoints obtained from sensitive and resistant. Unpaired t test, t_36_ = −1.143, P = 0.161, n = 19, respectively. **L** Pain threshold obtained from sensitive and resistant in hot plate test. Mann-Whitney Rank Sum Test, T = 344.5, P = 0.281, n = 19, respectively. Data are presented as mean values ± Interquartile range.

We expressed channelrhodopsin-2 (ChR2) bilaterally in dopaminergic neurons of the VTA and implanted an optic fibre that was aimed at the VTA (oICSS mice, Fig. 1B, C, D). Training was performed 3 weeks after virus expression. The training method is shown in Fig. 1A. When a mouse responds to the active poke, resulting in VTA stimulation, laser stimulation (15-ms pulse width at 20 Hz) with 5 s cuelight. Then, mice received 3 days of 1 h punishment tests in which every third laser stimulus was paired with an additional foot shock (0.2 mA, 0.5 s). An unsupervised clustering analysis integrating three behavioral parameters (active nosepokes, futile nosepokes, and triggers) over the last two punishment sessions (P2 and P3) yielded two clusters: shock-resistant (persever) and shock-sensitive (renouncer) mice (Fig. 1E, F). Facing noxious footshock, sensitive mice reduced the number of laser acquisition quickly, while resistant mice continued to seek the laser stimulation regardless of the harmful footshock (Fig. 1G, H). The Laser stimulation acquisition behaviors and the number of active pokes were different between the two groups, suggesting that divergent responses to punishment may influenced by the history of oICSS. Inactive pokes during the acquisition sessions and the break points in the progressive ratio test (Fig. 1I, J) were similar in both groups, suggesting that the two groups of mice showed an equal intensity of desire and motivation for the laser-stimulated reward and were willing to make efforts for it. Moreover, there was no difference in pain threshold between the two groups of mice in the hot plate test (Fig. 1K), implying that different responses to punishment were not due to different pain sensitivity (Fig. 1L).

### The SC neurons were inhibited in persever mice

**Fig. 2:**
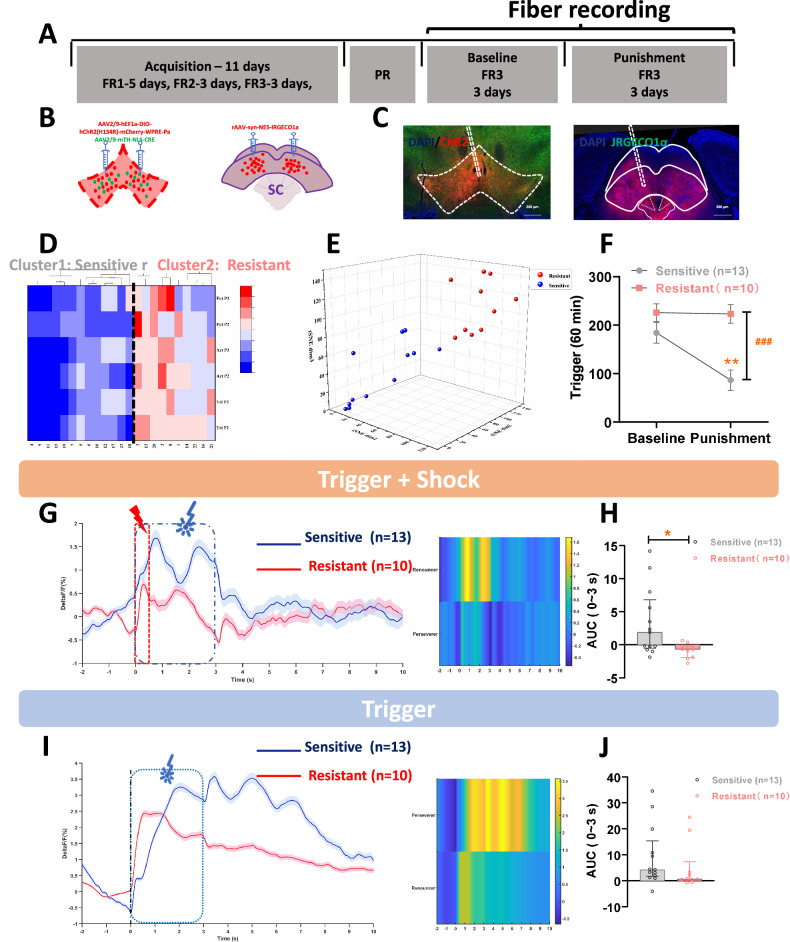
The SC neurons were inhibited in persever mice. **A** Experimental timeline for identifying mice with oICSS compulsive behavior. **B** Schematic of virus injection sites and optic fiber implantation sites. Scale bars, 200 μm. **C** ChR2-mCherry and JRGECO1a coronal slice. Scale bars, 200 μm. **D** Hierarchical clustering was performed based on the homogenization of different parameters of penalty sessions 2 and 3 (P2 and P3) of oICSS. **E** t-SNE three-dimensional representation of clusters of resistant (cluster 1) and sensitive (cluster 2) in oICSS. **F** Resistant and sensitive laser stimulation obtained from the baseline and the punishment session. Resistant had more laser stimulation compared with sensitive. Two-way analysis of variance (ANOVA) revealed a statistically significant interaction effect, F_1,45_ = 5.049, *P = 0.03, Holm-Sidak post hoc analysis, ###P < 0.001 for sensitive versus resistant in punishment, **P = 0.001 for punishment versus baseline in sensitive; n = 10–13, respectively. **G** Heatmap and plot of deltaF/F SC Ca2+ signals in resistant and sensitive groups during punishment. **H** AUC of deltaF/F SC Ca^2+^ signals in 0–3 s. Mann-Whitney Rank Sum Test, T = 81, *P = 0.017, n = 10–13, respectively. Data are presented as mean values ± Interquartile range. **I** Heatmap and plot of deltaF/F SC Ca^2+^ signals in resistant and sensitive groups during baseline. **J** AUC of deltaF/F SC Ca^2+^ signals in 0–3 s. Mann-Whitney Rank Sum Test, T = 92, P = 0.088, n = 10–13, respectively. Data are presented as mean values ± Interquartile range.

Next, we expressed JRGECO1α in SC neurons and recorded in vivo calcium signals during the punishment test to examine SC neuronal activity in two mice groups (Fig. 2A-C). The footshock test was performed after training, and shock-resistant (perseverer) and shock-sensitive (renouncer) mice (Fig. 2D, E) were successfully screened out. When facing noxious footshock, sensitive mice reduced the number of laser acquisition quickly. In contrast, resistant mice kept getting laser regardless of punishment (Fig. 2F). Fiber photometry recordings were applied in two time periods: pre-shock training and shock testing. In the plantar shock test, compared with shock-sensitive (renouncer) mice, the activity of SC neurons was inhibited when shock-resistant (perseverer) mice continually received laser stimulation and footshock in punishment test (Fig. 2G, H). However, during the baseline training before the foot-shock test, the excitability of SC neurons in both shock-resistant and shock-sensitive mice significantly increased upon laser stimulation (Fig. 2I), with no differences between the two groups (Fig. 2J). But 3 s after the laser appeared, the response of the sensitive mice to the laser was significantly different from the strong initial response of the resistant mice. The excitability of the SC neurons in the sensitive mice was higher than that in the resistant mice, which might reflect the differences in neural adaptability between the two groups. The continuously higher response of the sensitive mice might represent a healthy and regulated “motivational attention” maintenance, which supports the continuous anticipation and preparation for the upcoming reward. The resistant mice, on the other hand, might represent an early and excessive intervention of abnormal rapid, and active inhibitory mechanisms reflecting neural adaptability abnormalities. Together, these results suggest that laser self-stimulation under punishment is associated with overinhibition of neurons in the SC.

### Specific inhibition of SC neurons significantly enhances compulsive behavior in mice

**Fig. 3:**
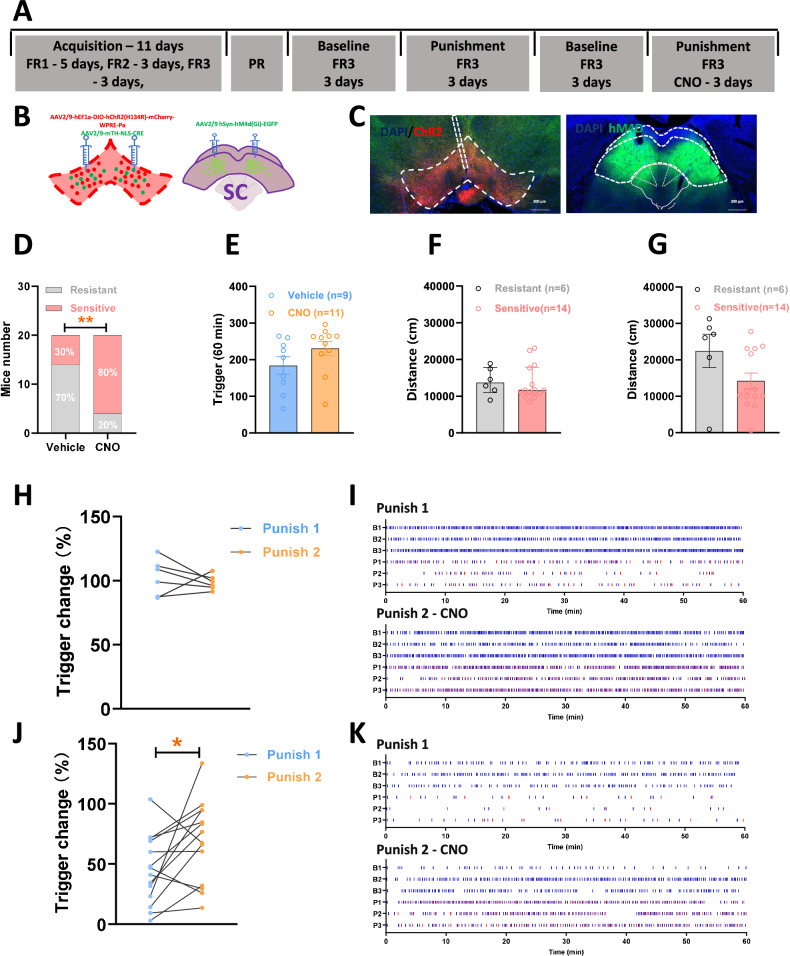
Effect of inhibition of SC neurons on the compulsive behavior of oICSS. **A** Timeline of the experiment. **B** Schematic of virus injection sites and optic fiber implantation sites. Scale bars, 200 μm. **C** ChR2-mCherry and hM4D coronal slice. Scale bars, 200 μm. **D** Ratio of in resistant and sensitive in AAV-hM4D group before and after SC inhibition; Fisher’s exact test, **P < 0.01. **E** hM4D mediated inhibition of aIC did not affect the positive reinforcement effect of the laser. T-test, t_18_ = −1.552, P = 0.138, n = 9–11, respectively. Data are presented as mean values ± SEM. **F** Locomotor activity in resistant and sensitive mice. Mann-Whitney Rank Sum Test, T = 71, P = 0.536, n = 9–11, respectively. Data are presented as mean values ± Interquartile range. **G** Locomotor activity was not affected by hM4D mediated inhibition of SC in resistant and sensitive mice. Unpaired t-test, t_18_ = 1.879, P = 0.076, n = 9–11, respectively. Data are presented as mean values ± SEM. **H** Inhibition of SC neurons did not affect compulsive behavior in resistant. Paired t-test, t_19_ = −1.783, P = 0.091, n = 20, respectively. Data are presented as mean values ± SEM. **I** Raster plots of laser stimulation (blue line) and punishment (red line) at baseline and during punishment before and after CNO intervention in resistant. **J** Inhibition of SC neurons enhances compulsive behavior in sensitive. Paired t-test, t_13_ = 2.381, *P = 0.033, n = 14, respectively. Data are presented as mean values ± SEM. **K** Raster plots of laser stimulation (blue line) and punishment (red line) at baseline and during punishment before and after CNO intervention for sensitive.

To determine the causal link between inhibited SC neuronal excitability and resistance to punishment, we inhibited SC neurons through inhibitory designer receptor exclusively activated by designer drug (DREADD) hM4D via bilateral infusion of rAAV-hSyn-hM4D(Gi)-EGFP in SC (Fig. 3A-C). For control group, AAV-hSyn-EGFP was bilaterally infused into SC. Training was performed 2 weeks after virus expression. After the third punishment test, 6 of 20 (≈30%) mice were identified as resistant in control group and 6 of 20 (30%) mice were resistant in experimental group. After 3 days of recovery training, we activated the inhibitory DREADD hM4Di in SC neurons via intraperitoneal administration of CNO (3 mg/kg) 15 min before each punishment test. After the sixth punishment test, 16 of 20 (80%) mice were identified as resistant mice in experimental group (Fig. 3D). In addition, after the inhibition of SC neurons, there was no change in the laser-induced positive reinforcement (Fig. 3E). There was no difference in the basic locomotor activity of resistant mice and sensitive mice (Fig. 3F), and still no difference was observed after inhibiting the SC neurons (Fig. 3G). This suggests that the increase in compulsive behavior was not due to changes in laser-induced positive reinforcement or locomotor activity. Chemogenetic inhibition of SC among resistant (perseverer) mice did not affect compulsive behavior in the punishment test (Fig. 3H). Figure 3I represents the raster plots for laser (blue lines) and punishments (red lines) in baseline and punishment sessions of resistant (perseverer) mouse before and after the inhibition of SC neurons. In contrast, inhibition of SC elicited a significant increase of compulsive behavior among sensitive (renouncer) mice, and sensitive mice decreased sensitivity to punishment (Fig. 3J). Figure 3K represents the raster plots for laser (blue lines) and punishments (red lines) in baseline and punishment sessions of sensitive (renouncer) mouse before and after the inhibition of SC neurons.

### Specific activation of SC neurons significantly suppresses compulsive behavior in mice

**Fig. 4:**
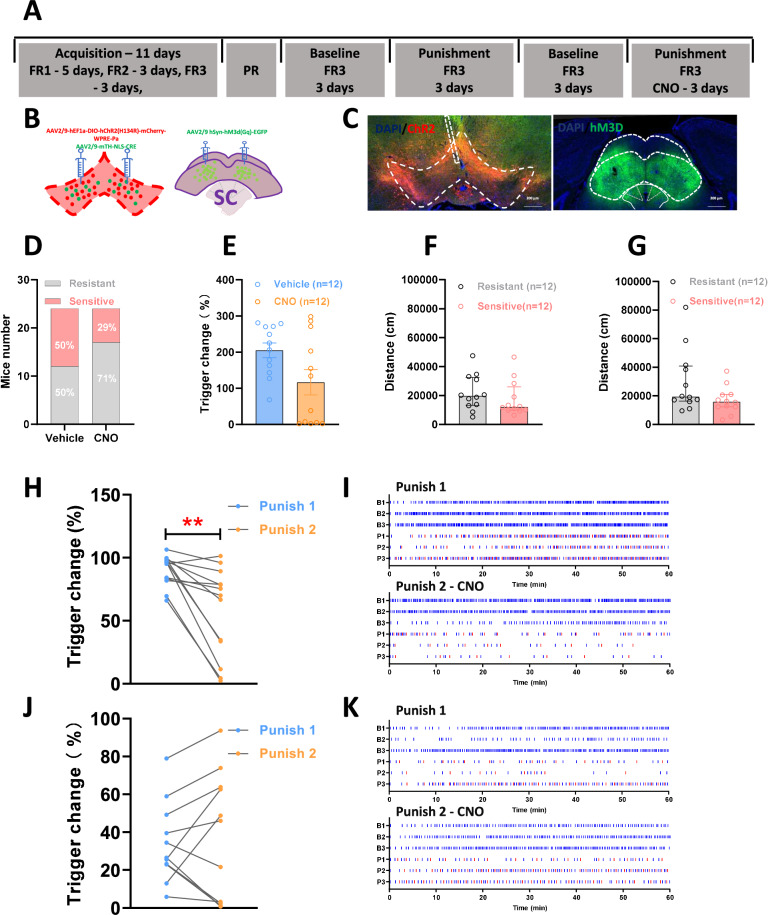
Effect of activation of SC neurons on the compulsive behavior of oICSS. **A** Timeline of the experiment. **B** Schematic of virus injection sites and optic fiber implantation sites. Scale bars, 200 μm. **C** ChR2-mCherry and hM3D coronal slice. Scale bars, 200 μm. **D** Ratio of in resistant and sensitive in AAV-hM3D group before and after SC inhibition. **E** hM3D mediated inhibition of aIC did not affect the positive reinforcement effect of the laser. Mann-Whitney Rank Sum Test, T = 178, P = 0.106, n = 12, respectively. Data are presented as mean values ± Interquartile range. **F** Locomotor activity in resistant and sensitive mice. Mann-Whitney Rank Sum Test, T = 147, P = 0.237, n = 12, respectively. Data are presented as mean values ± Interquartile range. **G** Locomotor activity was not affected by hM3D mediated activation of SC in resistant and sensitive mice. Unpaired t-test, t_22_ = 1.876, P = 0.074, n = 12, respectively. Data are presented as mean values ± SEM. **H** Activation of SC neurons decreases compulsive behavior in persistent individuals. Paired t-test, t_12_ = 3.892, **P = 0.002, n = 13, respectively. Data are presented as mean values ± SEM. **I** Raster plots of laser stimulation (blue line) and punishment (red line) at baseline and during punishment before and after CNO intervention in resistant. **J** Activation of SC neurons did not affect compulsive behavior in sensitives. Paired t-test, t_10_ = −0.542, P = 0.6, n = 11, respectively. Data are presented as mean values ± SEM. **K** Raster plots of laser stimulation (blue line) and punishment (red line) at baseline and during punishment before and after CNO intervention for sensitive.

To study whether activation of SC neurons was sufficient to reduce compulsive behavior, AAV-hSyn-hM3D-EGFP was infused into SC, allowing for the incorporation of excitatory DREADD in SC neurons (Fig. 4A-C). For control group, AAV-hSyn- EGFP was infused into SC. Training was performed 2 weeks after virus expression. After the third punishment test, 12 of 24 (50%) mice were identified as resistant in the experimental group. After 3 days of recovery training, we activated the excitatory DREADD hM3Dq in SC neurons via intraperitoneal administration of CNO (3 mg/kg) 15 min before each punishment test. After the sixth punishment test, 7 of 24 (29%) mice were identified as resistant mice in experimental group (Fig. 4D). In addition, after the activation of SC neurons, there was no change in the laser-induced positive reinforcement (Fig. 4E). There was no difference in the basic locomotor activity of resistant mice and sensitive mice (Fig. 4F), and still no difference was observed after activating the SC neurons (Fig. 4G). These suggest that the decrease in compulsive behavior was not due to changes in laser-induced positive reinforcement or locomotor activity. Activation of SC elicited a significant decrease of compulsive behavior among resistant (perseverer) mice; resistant mice restored sensitivity to punishment (Fig. 4H). Figure 4I represents the raster plots for laser (blue lines) and punishments (red lines) in baseline and punishment sessions of resistant (perseverer) mouse before and after the activation of SC neurons. In contrast, chemogenetic activation of SC among sensitive (renouncer) mice did not affect laser stimulation in the punishment test (Fig. 4J). Figure 4K represents the raster plots for laser (blue lines) and punishments (red lines) in baseline and punishment sessions of resistant (perseverer) mouse before and after the inhibition of SC neurons.

## Discussion

In this study, mice with shock-resistant phenotype (compulsive behavioral phenotype, perseverer) were successfully screened based on the mouse oICSS model. Our study reveals the regulatory role of SC on compulsive drug use behavior during addiction and its underlying neural circuit mechanism, showing that dynamic inhibition of SC neuronal activity is a core feature of the shock-resistant, and modulation of SC activity significantly alters individual behavioral response thresholds to negative aversive stimuli. Specifically, in the shock-resistant phenotype, activation of SC significantly reduced the number of laser acquisition in the punishment phase, while inhibition of SC had no change. In the shock-sensitive phenotype, SC inhibition significantly increased the number of laser acquisition during the punishment phase, while activation was unchanged. In addition, the number of laser acquisition during oICSS in the shock-resistant group was significantly penalty sensitive, but PR motivation and basal pain threshold did not differ between the two groups.

It is noteworthy that the optogenetically-induced oICSS paradigm, combined with plantar shock punishment established in this study, successfully recapitulates the phenotypic stratification observed in clinical addiction populations (20–50% compulsive phenotype). Compared to traditional drug self-administration models, this paradigm achieves specific optogenetic activation of VTA dopaminergic neurons independent of direct pharmacological action, thereby effectively decoupling drug metabolic effects from neural circuit adaptations. This innovation provides a novel framework for isolating pure circuit mechanisms underlying addiction-related compulsive behaviors. In the oICSS model, we observed that the activity of the SC in the shock-resistant mice was significantly lower than that in the shock-sensitive mice. In this study, the main focus was on the deep brain regions of the SC. As the SC is a crucial visual-motor integration center, its deep neurons directly project to the VTA and the caudal unassigned zone (cZI), forming a multi-synaptic pathway that regulates reward-seeking and avoidance behaviors [51]. Low SC activity may weaken its excitatory drive to downstream punishment-related brain regions (such as the lateral habenula or periaqueductal gray), thereby reducing the processing of avoidance signals triggered by electric shock [52]. Meanwhile, SC enhances the reward motivation by activating midbrain dopaminergic neurons (such as VTA) [34]. The low activity of SC in mice may reflect the dominant regulation of its reward pathway - even in the face of punishment, the reward drive still prevails, leading to persistent compulsive light-seeking behavior.

In our study, mice were divided into shock-resistant and shock-sensitive groups based on their performance in the punishment test. This classification method was influenced by the factor that the mice had previously received rewards from the “unconditioned stimulus-response system”. Because the “unconditioned stimulus-response system” rewards that easily frightened mice received at the baseline stage were significantly less than those that were resistant to fear. Still, there was no difference in motivation between the two groups of mice in the PR motivation test. Motivational differences constitute one factor contributing to the development of compulsivity, but not the sole determinant. The progression from regular drug use to compulsive behavior in addiction reflects a shift in the animal’s behavioral pattern from goal-directed to habitual responding1. The differences in operant response rates under the fixed-ratio (FR) baseline schedule may indicate early divergence in behavioral execution patterns or impulsivity between the two groups [53–55]. A higher baseline response rate may reflect a more stereotyped, automated (i.e., habitual) seeking pattern, which itself represents a key behavioral characteristic in the development of compulsive behavior [56]. In other words, shock-resistant mice already exhibit a stronger tendency toward habitual responding even in the absence of punishment, a tendency that may manifest as reduced sensitivity to cost signals and greater difficulty in behavioral inhibition when punishment is introduced. Although the two groups differed in their baseline fixed ratio (FR) response rates, they showed no significant difference in the breakpoint reached during the progressive-ratio (PR) test. The PR test is a standard paradigm for assessing the maximum effort an animal is willing to expend to obtain a reward—that is, its motivational strength or reward valuation [57–60]. This result strongly suggests that the two groups possess comparable levels of intrinsic drive or “wanting” for the optical reward. Therefore, the behavioral divergence observed during the punishment phase cannot be simply attributed to an overall difference in reward motivation. Moreover, in existing published studies on addiction-related compulsivity, motivation levels are consistently reported as similar between shock-resistant and shock-sensitive animals [3, 5, 6, 61].

The functional heterogeneity of the SC in compulsive behaviors may be closely linked to its multimodal information integration properties. Previous studies have established the SC’s central roles in visual threat detection, innate instinctual behavior modulation, and spatial attentional allocation [7–9, 26, 62, 63]. This study revealed that shock-resistant mice exhibited a stage-specific reduction in SC neuronal excitability during phases of compulsive drug-taking. Using fiber-optic recordings, we observed that in oICSS mice, SC neurons showed significantly reduced excitability and decreased responsiveness to aversive stimuli, such as foot shocks. These findings suggest that addiction may reshape SC neuroplasticity [64], thereby impairing its ability to encode the salience of aversive stimuli. Specifically, repeated drug intake may lead to changes in synaptic strength and neurotransmitter systems within the SC, such as reduced GABAergic inhibition or enhanced glutamatergic transmission, resulting in its hypoexcitatory state [33]. Such adaptive alterations could potentially drive reward-punishment weight imbalance, predisposing individuals to excessive reward-seeking bias during approach-avoidance decision-making. In the context of addiction, the disrupted SC function may disrupt the normal balance between positive reinforcement (reward) and negative reinforcement (punishment), leading to a preference for drug-related rewards despite adverse consequences. This aligns with the dual-process theory of addiction, which posits that dysregulation of the brain’s reward and stress systems contributes to compulsive drug use [65, 66].

During compulsive reward-seeking behavior, a pathological positive feedback loop characterized by “disinhibition and enhanced drive” may exist between the hypoactivity of the SC and DA release in the VTA. When aversive stimuli (such as footshock) are present, the functional heterogeneity across SC layers, combined with its role as a key subcortical integration hub, may lead to reduced SC activity. This decline could directly or indirectly weaken inhibitory regulation over the mesolimbic dopamine system via relevant neural circuits, resulting in dysregulated reward signal processing and subsequent aberrant DA release [51]. Such dysregulated dopaminergic signaling may further reinforce stereotypic seeking behaviors and potentially impair value-based decision-making [67]. Concurrently, sustained dopaminergic drive may, through changes in synaptic plasticity, induce long-term suppression of SC function, thereby forming a vicious cycle that perpetuates and exacerbates compulsive behavior [68, 69].

Currently, research on the direct involvement of the SC in reward anticipation typically relies on explicit, spatiotemporally precise sensory cues—particularly visual cues—to trigger orienting and reward-seeking behaviors [26, 51, 70, 71]. However, in our baseline oICSS paradigm, manipulating the SC did not affect behavior. This may be because animals engaged in self-stimulation in the absence of external predictive cues, with their behavior primarily driven by internal motivational states and well-consolidated operant habits. We speculate that SC regulation of the mesolimbic system may play a more critical role in cue-triggered reward-seeking rather than in sustaining cue-free, persistent motivational states, and that this behavior relies on specific neuronal subtypes, particularly glutamatergic neurons [70]. The SC is known to evaluate the salience of external information effectively, participate in regulating approach–avoidance behavioral processes, and serve as a key nucleus for responding to aversive stimuli [14, 17, 39, 72]. During the baseline phase, animals did not experience any punishment; thus, SC manipulation did not affect baseline behavior. In contrast, during the punishment phase, when an aversive stimulus (foot shock) was present, SC manipulation altered the organism’s response to the negative stimulus. We recorded transient increases in SC activity at the “triggering” moment, which likely reflects instantaneous neural events related to the decision-making, initiation, and execution of individual operant actions (nose-pokes). Chemogenetic approaches produce slow, non-time-locked modulation of SC activity over several hours. Neural control of behavior is highly time-specific; prolonged alterations in activity may not mimic or interfere with transient activity patterns time-locked to specific behavioral moments [73]. Therefore, the absence of behavioral effects during the baseline period does not negate the functional role of SC activity at the “triggering” moment; rather, it underscores that the SC may act as a gate at the precise moment of action initiation, rather than setting the overall motivational tone over longer timescales. In the future, we will conduct direct verification using time-limited optogenetic intervention.

Building on chemogenetic modulation results, we propose that the SC participates in addiction behavior regulation through two parallel pathways: one is the SC-ventral tegmental area (VTA) dopaminergic circuit mediating gain control of reward-prediction signals, and another is the SC-lateral habenula or amygdala pathway modulating attenuation processing of negative valence signals. SC has a direct projection to VTA, and the projection from VTA to NAc can regulate the release of dopamine in the nucleus accumbens. The nucleus accumbens is a key node of the brain’s reward system [74–76]. Possibly by altering the strength of SC-VTA predictions, SC can influence the magnitude of reward prediction error in the VTA, thus shaping motivation and reinforcement learning [77, 78]. Meanwhile, the SC-lateral habenula or amygdala pathways may play a role in processing negative emotions and aversive experiences [79, 80]. Dysfunction in these pathways could lead to impaired fear conditioning and reduced sensitivity to punishment, facilitating the development of compulsive drug-seeking behavior. This hypothesis exhibits theoretical convergence with recent findings on the gating role of SC-thalamocortical circuits in affective-motivational conflict resolution [6, 14].

As a midbrain tectal structure, the superior colliculus (SC), with its superficial anatomical positioning and extensive integration with subcortical multisensory inputs, may serve as an ideal neuromodulatory target for physical intervention technologies such as transcranial magnetic stimulation (TMS) or transcranial direct current stimulation (tDCS) [81]. Anatomically, the SC is located just beneath the occipital cortex, with a relatively shallow depth of approximately 2 - 3 cm from the scalp surface in humans [29, 82]. This advantageous positioning facilitates non-invasive modulation by external stimuli, minimizing the technical challenges associated with reaching deeper brain structures. The inhibition of SC activity in the compulsive phenotype suggests that enhancing the excitability of the SC may potentially restore the behavioral sensitivity to aversive stimuli, thereby inhibiting compulsive drug use. Compared to traditional nucleus accumbens (NAc)- or prefrontal cortex (PFC)-targeted interventions, this strategy demonstrates superior spatial specificity and reduced risks of cognitive side effects [83–87]. This hypothesis methodologically complements recent studies employing SC alternating bilateral sensory stimulation (ABS) to ameliorate alcohol-induced conditioned place preference [88] and promote sleep [89], thus providing theoretical foundations for designing cross-substance addiction intervention strategies targeting shared neural mechanisms.

## Conclusion

In conclusion, the present study elucidated the modulatory role of SC in compulsive behaviors associated with drug addiction. SC is involved in regulating compulsive behaviors related to drug addiction by integrating processing signals related to punishing negative stimuli. These findings not only expand the theoretical framework of the traditional mesolimbic dopamine system in addictive behavior, provide a new perspective for understanding the neural mechanisms of addictive behavior, but also open up innovative ways to develop precisely targeted neuromodulation therapies.

## Limitation

This study has several important limitations. Firstly, at the model level, the intracranial self-stimulation model employed primarily relies on direct activation of a single neural pathway, which differs significantly from traditional drug addiction involving multiple brain regions, multiple neurotransmitter systems, and complex somatic metabolic and withdrawal responses. Thus, it may not fully capture the pathological complexity of human addiction. Secondly, the complexity of neural circuits has not been sufficiently dissected. The SC itself comprises various functionally distinct neuronal subtypes, yet this study did not perform cell-type-specific manipulations; therefore, it remains unclear which specific neurons (e.g., GABAergic interneurons or specific projection neurons) mediate the observed effects. Moreover, the study did not simultaneously monitor network-level activities between the SC and its key upstream and downstream brain regions (such as the anterior cingulate cortex and thalamus), leaving the precise circuit mechanisms by which the SC influences behavior largely speculative. Finally, the study generally used only a single sex (mostly male) of mice, overlooking the potentially significant effects of sex on addiction susceptibility, neural adaptation, and SC circuit function, thereby greatly limiting the generalizability of the findings. These limitations indicate that the current conclusions require further validation and elaboration using addiction models more closely aligned with human clinical conditions, intervention techniques with higher spatiotemporal resolution, multidimensional behavioral assessments, and experimental designs that include both sexes.

## Data Availability

Data will be made available on request.
